# Optimizing Distributions for Associated Entropic Vectors via Generative Convolutional Neural Networks

**DOI:** 10.3390/e26080711

**Published:** 2024-08-21

**Authors:** Shuhao Zhang, Nan Liu, Wei Kang, Haim Permuter

**Affiliations:** 1 School of Information Science and Engineering, Southeast University, Nanjing 211189, China; shzhang@seu.edu.cn; 2 National Mobile Communications Research Laboratory, Southeast University, Nanjing 211189, China; nanliu@seu.edu.cn; 3 Department of Electrical and Computer Engineering, Ben-Gurion University of the Negev, Beersheba 8410501, Israel; haimp@bgu.ac.il

**Keywords:** entropic vectors, entropic region, neural networks, convolutional neural networks, Ingleton score, Ingleton violation index, inner bounds, network coding

## Abstract

The complete characterization of the almost-entropic region yields rate regions for network coding problems. However, this characterization is difficult and open. In this paper, we propose a novel algorithm to determine whether an arbitrary vector in the entropy space is entropic or not, by parameterizing and generating probability mass functions by neural networks. Given a target vector, the algorithm minimizes the normalized distance between the target vector and the generated entropic vector by training the neural network. The algorithm reveals the entropic nature of the target vector, and obtains the underlying distribution, accordingly. The proposed algorithm was further implemented with convolutional neural networks, which naturally fit the structure of joint probability mass functions, and accelerate the algorithm with GPUs. Empirical results demonstrate improved normalized distances and convergence performances compared with prior works. We also conducted optimizations of the Ingleton score and Ingleton violation index, where a new lower bound of the Ingleton violation index was obtained. An inner bound of the almost-entropic region with four random variables was constructed with the proposed method, presenting the current best inner bound measured by the volume ratio. The potential of a computer-aided approach to construct achievable schemes for network coding problems using the proposed method is discussed.

## 1. Introduction

Given *n* discrete random variables, for a fixed joint distribution, all their $2^{n}-1$ (joint) entropies define an entropic vector in the entropy space $R^{2^{n}-1}$. By varying over all possible joint distributions, the set of all entropic vectors defines the entropic region. The entropic region plays a fundamental role in information theory and network coding. The closure, known as the almost-entropic region, yields rate regions for multi-source coded networks [1,2]. However, the complete characterization of the entropic region encounters challenges even when n=3 [3,4,5,6,7], where only the closure, i.e., the almost-entropic region, is fully characterized [3]. When n=4, the characterization of the almost-entropic region also becomes extremely difficult and remains open [8].

In the pursuit of the characterization of the entropic region, one crucial problem is to *determine whether an arbitrary vector in the entropy space is entropic or not*. If all entropic vectors in the entropy space are verified, the entropic region is fully characterized. Additionally, given a point in the rate region of a coded network, the existence of achievable codes relies on the underlying entropic vector [9].

This problem has been tackled from different perspectives in the literature. Information inequalities, which fully characterize all almost-entropic vectors, are shown to be infinitely many [10] and extremely hard to characterize [8,10,11,12,13,14,15,16,17]. The construction of entropic vectors, however, is more feasible and studied through probability distributions, e.g., [6,7,16,18,19,20,21,22,23,24,25,26,27,28], groups, e.g., [29,30,31,32,33,34,35], and matroids, e.g., [36,37,38,39,40]. Regarding the construction of entropic vectors from probability distributions, remarkable research has attempted to numerically obtain probability mass functions (PMFs) for entropic vectors [16,19,20,21,22,23,24,25,26,27], particularly when the alphabet sizes of the involved random variables are bounded. In this way, the entropic nature of a given vector in the entropy space is immediately verified following the definition if the underlying joint PMF is obtained.

Among these approaches, some are interested in quasi-uniform PMFs [19,27] or PMFs supported on small atoms [22,23,24,25], while more general PMFs are considered in [16,20,21,26]. For example, an algorithm is given in [20] to verify binary entropic vectors by recursively constructing PMFs. In [16], the PMFs are numerically obtained with Newton’s method. In [21], the study of the almost-entropic region when n=4 is reduced to a three-dimensional tetrahedron and visualized by various maximization procedures with different methods and strategies [21] (Section VII). Recently, a random search algorithm has been introduced [26] to find the nearest entropic vector to a given target vector. This algorithm iteratively tries a few random perturbations on PMFs, striving to decrease the normalized distance between an entropic vector and the target vector.

However, the limitations of the above methods cannot be overlooked. The method in [20] is only feasible for binary entropic vectors. In [16], and [21], formal algorithms and performances of described methods are not systematically discussed in detail. Although [26] presents more analytical and empirical results, the limitation of the randomized algorithm persists, and the convergence performances are not empirically guaranteed. Furthermore, a critical issue in above methods is that only PMFs with relatively small alphabet sizes can be handled. This limitation is vital, since the verification of an underlying entropic vector in the rate region of a coded network requires large alphabets when subpacketizations are necessary for achievable codes.

With these methods, which construct entropic vectors from PMFs, some interesting optimization problems over the entropic region when n=4 can be experimented. It is known that the Ingleton inequality [41] completely characterizes the part of the almost-entropic region achieved by linearly representable entropic vectors [13]; thus, it reduces the study to the entropic vectors violating it. To measure the degree of violation from the Ingleton inequality for an entropic vector, the *Ingleton score* [16,42] and *Ingleton violation index* [19] are proposed from different angles. Although remarkable efforts have been made to minimize the Ingleton score [16,21,26,35,42] and maximize the Ingleton violation index [19,26], their optimal values remain open. In [16], the Ingleton score is numerically optimized and conjectured to be −0.089373, known as the four-atom conjecture [16] (Conjecture 1). In [21], a technique is proposed to transform an entropic vector into another entropic vector, and the transformed vector is optimized to the Ingleton score −0.09243, which refutes the four-atom conjecture by [16]. By experimenting with groups [35], the best-known Ingleton score is currently −0.0925. The Ingleton violation index is optimized in [19] and, subsequently, in [43], and its best known value is currently 0.0281316, according to [26].

As the complete characterization of the almost-entropic region for n=4 is difficult, inner bounds can be constructed by taking the convex hull of certain acquired entropic vectors, as previously investigated in [16,22,26]. The quality of such an inner bound is measured by the volume ratio, i.e., the percentage of inner-bound polytope volume to the volume of the outer-bound polytope. In [16], several entropic vectors are optimized to form an inner bound with a volume ratio of 53.4815%. Using distributions supported on small atoms, [22] finds entropic vectors that yield an inner bound with the volume ratio 57.8%. In [26], with their proposed grid method, the current best-known volume ratio is 62.4774%, while the largest volume ratio, which requires the complete characterization of the almost-entropic region when n=4, is still open.

In this paper, we develop *a novel architecture for optimizing PMFs for associated entropic vectors via convolution neural networks (CNNs)*. Recently, applications of neural networks (NNs) can be found in information theory [44,45,46] and control theory [47,48]. Our motivation arises from the fruitful research on applying NNs to problems in information theory [44,45,46]. More specifically, in [45], a unique mapping that generates conditional PMFs is defined and approximated using NNs. In [46], a model which generates a capacity-achieving input PMF for a given channel in discrete input spaces is proposed. Consequently, we are especially interested in approximating another mapping, which generates joint PMFs for multiple random variables. Sharing similar ideas with [26], the problem of verifying an entropic vector can be performed by minimizing the normalized distance through NN training. Furthermore, we are motivated to modify NNs due to the special structures and complexities of joint PMFs. Additionally, the developed method can be applied immediately to the optimizations of the Ingleton score and Ingleton violation index, and the construction of inner bounds for the almost-entropic region.

The major contributions of this paper are summarized as follows.

- A novel algorithm is proposed to optimize distributions for entropic vectors with NN training. For each target vector, an NN is trained such that the output PMF produces an entropic vector as close to target as possible. In practice, we implement the algorithm with CNNs, which accelerate and enable the algorithm to generate PMFs with large alphabets.
- The effectiveness of our proposed method is verified by empirical results. More specifically, smaller normalized distances and improved convergence performances are achieved by our proposed method, compared to [26]. In addition, with derived theoretical guarantees, by exploiting the proposed algorithm, the state-of-the-art Ingleton score is reconfirmed, and a new tighter lower bound of the Ingleton violation index is obtained. Furthermore, by utilizing the proposed algorithm, we develop another algorithm to construct a new inner bound of the almost-entropic region (n=4), yielding the current best inner bound measured by the volume ratio.

This paper is organized as follows. Section 2 introduces preliminaries, notations, and the problem statement. The proposed method and algorithm with derived theoretical guarantees are presented in Section 3, and the implementation with CNNs is demonstrated in Section 4. In Section 5, empirical results exploiting the proposed method for several problems when n=4 are presented. Section 6 summarizes the paper in general, and discusses the potential of the proposed method to construct achievable schemes for network coding problems.

## 2. Preliminaries and Problem Statement

In this section, we provide the preliminaries, notations, and the problem statement of this paper.

### 2.1. Convex Cones and Convex Polytopes

Given a set $C\subset R^{d}$, *C* is a *pointed cone* if a∈C implies that ta∈C for all real t≥0, and $R_{a}=\left\{ta,t\in R^{+}\right\}\subset C$ is a *ray* of *C*. Pointed cones are unbounded except {0}, where 0 is the origin of Euclidean space. In the rest of the paper, all cones are assumed to be pointed and unbounded. For all real t≥0, $R_{b}=\left\{tb\right\}$ of *C* is an *extreme ray* if b cannot be expressed as the positive linear combination of any $a_{1},a_{2}\in C$. A cone *C* is convex if *C* is a convex set.

Given a convex set $C\subset R^{d}$, *C* is a *convex polyhedron* if it is the intersection of finitely many halfspaces (i.e., linear inequalities). A convex cone *C* is a special polyhedron when the halfspaces that define *C* contain 0 simultaneously, and, in this case, the convex cone *C* is called *polyhedral*. A convex polyhedron is a *convex polytope* if it is bounded. In the rest of the paper, all polytopes are assumed to be convex. For a convex cone (or convex polytope), if we specify one extreme ray (or vertex) as the *top* and others as the *bases*, then the convex cone (or convex polytope) is often called the *pyramid*.

For a convex polyhedron *C*, there are two equivalent types of representations, i.e., the *H-representation* and the *V-representation* [49] (Chapter 1). The H-representation is the set of all halfspaces defining *C*, and the V-representation is the set of all vertices (if *C* is a convex polytope) or extreme rays (if *C* is a polyhedral convex cone) defining *C*. The transformation between the H-representation and the V-representation for a given convex cone or convex polytope can be numerically performed with the implementation [50,51,52] of the double description method [53]. More details about convex cones and convex polytopes can be found in [49].

### 2.2. Entropic Vectors and Entropic Region

Let n≥2, consider a discrete random vector $X≜(X_{1},X_{2},\ldots ,X_{n})$ with a finite index set $N_{n}=\left\{1,2,\ldots ,n\right\}$, and X takes values in the finite alphabet $X≜X_{1}\times X_{2}\times \ldots \times X_{n}$ of size $\left|X\right|=m=\prod_{i=1}^{n}m_{i}$. The realization of X is denoted as x. Let p(x) denote the probability of x such that p(x)=Pr{X=x}. The joint probability mass function (PMF) of X is denoted as $p≜{\left[p\left(x\right),x\in X\right]}^{T}\in \Delta_{X}^{m}$, where $\Delta_{X}^{m}$ is the *m*-dimensional probability simplex defined as

(1) $$\Delta_{X}^{m}≜\left\{p\in R^{m}|\sum p\left(x\right)=1,p\left(x\right)\geq 0,\forall x\in X\right\}.$$

A vector in $R^{m}$ is a *valid PMF* if it belongs to the set $\Delta_{X}^{m}$.

We consider a subset of components of the random vector X; the above quantities can be denoted accordingly. More specifically, we consider the set of random variables $X_{\alpha }≜\left\{X_{i},i\in \alpha \right\},\alpha \subseteq N_{n}\setminus \emptyset$, which takes values in the finite alphabet $X_{\alpha }=\prod_{i\in \alpha }X_{i}$. The realization of $X_{\alpha }$ is denoted as $x_{\alpha }$. Let $p_{\alpha }\left(x_{\alpha }\right)$ denote the probability of $x_{\alpha }$ such that $p_{\alpha }\left(x_{\alpha }\right)=\mathrm{Pr}\left\{X_{\alpha }=x_{\alpha }\right\}$. The marginal PMF of $X_{\alpha }$ is denoted as $p_{\alpha }≜{\left[p_{\alpha }\left(x_{\alpha }\right),x_{\alpha }\in X_{\alpha }\right]}^{T}$. Then, the Shannon entropy of $X_{\alpha }$ is

(2) $$H\left(X_{\alpha }\right)=-\sum_{x_{\alpha }\in X_{\alpha }}p_{\alpha }\left(x_{\alpha }\right)\log_{2}p_{\alpha }\left(x_{\alpha }\right),\;\forall \alpha \subseteq N_{n}\setminus \emptyset ,$$

where $0\log_{2}0≜0$. We define $h_{\alpha }≜H\left(X_{\alpha }\right)$, which is the *entropy function*. The vector consisting of entropy functions for all $\alpha \subseteq N_{n}\setminus \emptyset$ is the *entropic vector*, as formally defined in the following definition.

**Definition** **1** (Entropic vectors [54], Chapter 13)**.** *For the random vector X and index set $N_{n}$, given a joint PMF $p\in \Delta_{X}^{m}$, the entropic vector is defined as*

(3) $$\begin{array}{c} h≜{\left(h_{\alpha },\alpha \subseteq N_{n}\setminus \emptyset \right)}^{T}, \end{array}$$

*and h is the associated entropic vector of p, denoted as $h^{p}$.*

We note that, by varying α, there are total $2^{n}-1$ (joint) entropies for the random vector X. Thus, each h can be viewed as a vector in the $\left(2^{n}-1\right)$-dimensional Euclidean space, which is defined as the *entropy space*

(4) $$H_{n}≜R^{2^{n}-1},\;n\geq 2.$$

For example, given a random vector $X=(X_{1},X_{2},X_{3},X_{4})$, let each random variable in X be uniformly i.i.d.-distributed on alphabet {0,1}; then, the associated entropic vector in the entropy space $H_{4}=R^{15}$ is

(5) $$\begin{array}{cc} h^{p} & ={\left(h_{1},h_{2},h_{3},h_{4},h_{12},h_{13},h_{14},h_{23},h_{24},h_{34},h_{123},h_{124},h_{134},h_{234},h_{1234}\right)}^{T}.\\ \; & ={\left(1,1,1,1,2,2,2,2,2,2,3,3,3,3,4\right)}^{T}. \end{array}$$

Allowing infinite alphabets, the region in $H_{n}$ consisting of all entropic vectors is the *entropic region* ([54], Chapter 13)

(6) $$\Gamma_{n}^{*}≜\left\{h\in H_{n}|\;h\;\mathrm{is}\;\mathrm{entropic}\;\mathrm{vector}\right\}.$$

The closure of $\Gamma_{n}^{*}$ is the *almost-entropic region*${\bar{\Gamma }}_{n}^{*}$ ([54], Chapter 15).

Given a set of random variables, *basic inequalities* form the set of inequalities implied by the nonnegativity of all Shannon’s information measures. The nonredundant subset of basic inequalities consists of *elemental inequalities* ([54], Chapter 14), which are defined as the following two types of inequalities for the random vector X

(7) $$\begin{array}{cc} H(X_{i}|X_{N_{n}\setminus \left\{i\right\}}) & \geq 0,\;i\in N_{n}, \end{array}$$

(8) $$\begin{array}{cc} I(X_{i};X_{j}|X_{K}) & \geq 0,\;i\neq j,K\subset N_{n}\setminus \left\{i,j\right\}. \end{array}$$

The inequalities implied by elemental inequalities are *Shannon-type inequalities*. The region in $H_{n}$ that consists of vectors satisfying all elemental inequalities is ([54], Chapter 14)

(9) $$\Gamma_{n}≜\left\{h\in H_{n}|\;h\;\mathrm{satisfies}\;(7)\;\mathrm{and}\;(8)\right\}.$$

Sometimes $\Gamma_{n}$ is called the *polymatroidal region* because of the equivalence between the elemental inequalities for vectors in the entropy space and the polymatroidal axioms for polymatroidal rank functions [55].

Although both $\Gamma_{n}^{*}$ and $\Gamma_{n}$ are regions within $H_{n}$, the former stands for the associated entropic vectors defined by valid PMFs, while the latter is formed by vectors satisfying all Shannon-type inequalities. Hence, it is reasonable to question the identity between $\Gamma_{n}^{*}$ and $\Gamma_{n}$. It was first discovered by [3,8] that $\Gamma_{n}$ is a loose outer bound of $\Gamma_{n}^{*}$. More specifically, it is known that $\Gamma_{2}^{*}=\Gamma_{2}$, $\Gamma_{3}^{*}\neq \Gamma_{3}$ but ${\bar{\Gamma }}_{3}^{*}=\Gamma_{3}$, and ${\bar{\Gamma }}_{n}^{*}\subset \Gamma_{n}$ when n≥4 [3,8]. These relations reveal that there are inequalities tighter than Shannon-type inequalities as the outer bound of the entropic region, i.e., the inequalities hold for all entropic vectors but cannot be implied by elemental inequalities, and these inequalities are often referred to as the *non-Shannon-type inequalities* ([54], Chapter 15).

We briefly introduce some known structures of $\Gamma_{n}^{*}$, ${\bar{\Gamma }}_{n}^{*}$ and $\Gamma_{n}$. Both $\Gamma_{n}$ and ${\bar{\Gamma }}_{n}^{*}$ are pointed convex cones in the nonnegative orthant of $H_{n}$ [54] (Chapter 13). The region $\Gamma_{n}$ is polyhedral, while ${\bar{\Gamma }}_{n}^{*}$ is not when n≥4 [10], i.e., the convex cone $\Gamma_{4}$ (${\bar{\Gamma }}_{4}^{*}$) is represented with finitely many (infinitely many) extreme rays or finitely many (infinitely many) linear inequalities. For n≥3, although the almost-entropic region ${\bar{\Gamma }}_{n}^{*}$ is a convex cone, the region $\Gamma_{n}^{*}$ is not convex and $\Gamma_{n}^{*}\subset {\bar{\Gamma }}_{n}^{*}$. The complete characterization of ${\bar{\Gamma }}_{n}^{*}$ for n≥4 is difficult and open. For more details of $\Gamma_{n}^{*}$, ${\bar{\Gamma }}_{n}^{*}$, and $\Gamma_{n}$, please refer to [54] (Chapter 13–15).

Given the difficulty of characterizing $\Gamma_{n}^{*}$ with infinite alphabets, in order to optimize finite PMFs numerically, it is practical to consider $\Gamma_{n}^{*}$ when the random variables have finite alphabet sizes.

**Definition** **2** (Alphabet-bounded entropic region [54], Chapter 21)**.** *Given a random vector X with an index set $N_{n}$, taking values on the finite alphabet X of size $m=\prod_{i=1}^{n}m_{i}$ such that m<∞, the alphabet-bounded entropic region is defined as*

(10) $$\Gamma_{n,X}^{*}≜\left\{h^{p}\in H_{n}|\;p\in \Delta_{X}^{m}\right\},$$

*where $h^{p}$ is the entropic vector associated with the PMF p, and $\Delta_{X}^{m}$ is the probability simplex defined on the finite alphabet X of size m.*

The region $\Gamma_{n,X}^{*}$ is the collection of entropic vectors associated with PMFs defined on the finite alphabet X, and is a compact and closed set.

To characterize the closeness of two given vectors, the most straightforward measurement is the angle between the rays determined by them. Here, we adopt the measure of *normalized distance*, proposed in [26].

**Definition** **3** (Normalized distance)**.** *Given vectors $a,b\in \Gamma_{n}\setminus \left\{0\right\}$, the normalized distance between a and b is defined as*

(11) $$d_{\mathrm{norm}}\left(a,b\right)=\frac{∥a-b^{\prime }∥}{∥b^{\prime }∥},$$

*where ∥·∥ is the $l_{2}$ norm, and $b^{\prime }≜\arg\inf_{\tilde{b}\in R_{b}}∥a-\tilde{b}∥$.*

We note that the normalized distance is the tangent of the angle between the rays determined by a and b. Thus, we also have

(12) $$d_{\mathrm{norm}}\left(a,b\right)=\frac{∥a-\frac{a\cdot b}{{∥b∥}^{2}}b∥}{∥\frac{a\cdot b}{{∥b∥}^{2}}b∥}.$$

### 2.3. Neural Networks

In general, an NN is a computational model, with significant expressive capability for desired functions [56]. Following convention, we now formally define fully connected feedforward multilayer NNs as a family of functions.

**Definition** **4** (Neural networks [56])**.** *With l∈N hidden layers of sizes $d_{1},d_{2},\ldots ,d_{l}\in N$, fixed input dimension $d_{0}$ and output dimension $d_{l+1}$, where $d_{0},d_{l+1}\in N$, let ∘ denote the composition of functions, a fully connected feedforward multilayer NN is defined as the following family of functions*

(13) $$G_{l}^{\left(d_{0},d_{l+1}\right)}≜\left\{g:R^{d_{0}}\to R^{d_{l+1}}|g\left(x_{0}\right)=f_{l+1}\circ \sigma \circ f_{l}\circ \cdots \circ \sigma \circ f_{1}\left(x_{0}\right)\right\},$$

*where, for j∈{1,2,…,l+1}, $f_{j}:R^{d_{j-1}}\to R^{d_{j}}$ is a linear operation*

(14) $$f_{j}\left(x_{j-1}\right)≜W_{j}x_{j-1}+b_{j},$$

*with the weight matrices $W_{j}\in R^{d_{j}\times d_{j-1}}$, the data vectors $x_{j-1}\in R^{d_{j-1}}$, and the bias vectors $b_{j}\in R^{d_{j}}$. The nonlinear activation function σ(·) is performed on vectors element-wise, such that, for j∈{1,2,…,l},*

(15) $${\left(x_{j}\right)}_{i}={\left[\sigma \left(f_{j}\left(x_{j-1}\right)\right)\right]}_{i},\;i=1,2,\ldots ,d_{j}.$$

*For an arbitrary number of hidden layers and sizes, fully connected feedforward multilayer NNs with the same fixed input and output dimensions are defined as*

(16) $$G^{\left(d_{0},d_{l+1}\right)}≜\underset{l\in N}{⋃}G_{l}^{\left(d_{0},d_{l+1}\right)}.$$

The set of all possible weights and biases of an NN is the *parameter space*, which is denoted as $\Phi \subset R^{d}$ (here, *d* is defined as the dimension of the parameter space). We sometimes denote an NN function $g\in G^{\left(d_{0},d_{l+1}\right)}$ with parameters ϕ∈Φ as $g_{\phi }$. Exploiting NNs, desired functions can be *parameterized* with ϕ, and *approximated* by optimizing ϕ with gradient descent methods [57]. Multilayer feedforward NNs are known to be universal approximators for any measurable function as long as the scale of the model is sufficiently large [56].

Activation functions of NNs may vary in forms for different tasks. More specifically, let the input be $y\in R^{d_{y}}$ with elements $y_{j},j\in \left\{1,2,\ldots ,d_{y}\right\}$; the most common activation functions include the *sigmoid* activation function $\sigma_{s}{\left(y\right)}_{j}=\frac{1}{1+\exp(-y_{j})}$ and the *ReLU* activation function $\sigma_{R}{\left(y\right)}_{j}=\max\left(y_{j},0\right)$. There are many variations of ReLU, including the *ELU* activation function $\sigma_{E}{\left(y\right)}_{j}=\left\{\begin{array}{cc} y_{j} & ,y_{j}\geq 0,\\ e^{y_{j}}-1 & ,y_{j}<0. \end{array}\right.$ A special activation function is the *softmax* activation function or the *softmax layer*, which is defined as $\sigma_{\mathrm{sm}}\left(\cdot \right):R^{d_{y}}\to R^{d_{y}}$, where

(17) $$\sigma_{\mathrm{sm}}{\left(y\right)}_{j}=\frac{\exp(y_{j})}{\sum_{j=1}^{d_{y}}\exp\left(y_{j}\right)},\;j=1,2,\ldots ,d_{y}.$$

We note that the output vector of the softmax layer has special properties, i.e., $\sigma_{\mathrm{sm}}{\left(y\right)}_{j}\geq 0$ and $\sum_{j=1}^{d}\sigma_{\mathrm{sm}}{\left(y\right)}_{j}=1$, which make the output vector a valid PMF in (1).

For details on NNs, please refer to [58] (Chapter 20).

### 2.4. Problem Statement

In this paper, when a target vector $h_{t}\in \Gamma_{n}$ is given, we are interested in finding an entropic vector $h^{p}$ in $\Gamma_{n,X}^{*}$, such that $d_{\mathrm{norm}}\left(h^{p},h_{t}\right)$ is relatively small. If $h_{t}$ is entropic, we aim to give the underlying PMF to verify and realize $h_{t}$. If $h_{t}$ is not entropic, we aim to give an entropic vector close to $h_{t}$ and, hopefully, on the boundary of $\Gamma_{n,X}^{*}$.

We recall that $d_{\mathrm{norm}}\left(h^{p},h_{t}\right)$ is the tangent of the angle between the corresponding rays, but the tangent is only strictly increasing when the angle is limited in $\left[0,\frac{\pi }{2}\right]$. Restricting the target vector $h_{t}$ to be in $\Gamma_{n}$ can satisfy this requirement, since $\Gamma_{n}$ belongs to the nonnegative orthant of $H_{n}$ and $\Gamma_{n,X}^{*}\subset \Gamma_{n}$.

## 3. Optimizing PMFs for Associated Entropic Vectors via Neural Networks

In this section, we propose the methodology that tackles the problem stated in Section 2.4. More specifically, given a target vector in $\Gamma_{n}$, we propose to train a corresponding NN to identify the entropic vector closest to the target, and, at the same time, provide the corresponding underlying PMF.

In particular, the proposed NN is configured with a final softmax layer to output PMFs, with the input fixed to a specific constant (similar settings can be found in [45,46]). We claim that, for a given target vector, there always exists an NN such that the entropic vector associated with the produced PMF is arbitrarily close to the target in terms of normalized distance. This statement is formalized in the following theorem.

**Theorem** **1.** *For the random vector X with a fixed and finite alphabet X, given a target $h_{t}\in \Gamma_{n}$ (entropic or not), we consider the entropic vector $h^{*}\in \Gamma_{n,X}^{*}$ that yields the minimal normalized distance $D^{*}$ to $h_{t}$. Then, for η>0, there exists a corresponding NN $g_{\phi }$ with parameters ϕ∈Φ (where $\Phi \subset R^{d}$ is the parameter space) which outputs the PMF $p^{\phi }$ valid in $\Delta_{X}^{m}$ from a specific constant input a∈R, such that*

(18) $$d_{\mathrm{norm}}\left(h^{p^{\phi }},h_{t}\right)<D^{*}+\eta ,$$

*where $h^{p^{\phi }}$ is the associated entropic vector of $p^{\phi }$.*

The proof of Theorem 1 is straightforward based on the observation that the softmax layer can produce any desired PMF, and both the entropic vector and the normalized distance are continuous functions of the PMF. However, for the completeness of the paper, we provide the proof of Theorem 1 in Appendix B.1. The proof can be described as follows. For a given target vector $h_{t}\in \Gamma_{n}$, let $p^{*}$ be one of the underlying PMFs that achieves $h^{*}$, i.e., the entropic vector with minimal normalized distance to $h_{t}$; then, we can find an NN which outputs $p^{\phi }$ from a fixed constant, and $p^{\phi }$ approximates $p^{*}$ in $l_{1}$ norm. By the continuity of the Shannon entropy ([54], Section 2.3), for fixed finite alphabets, we can prove that, as $p^{\phi }$ approximates $p^{*}$ in $l_{1}$ norm, the associated entropic vector $h^{p^{\phi }}$ approximates $h^{*}$ in normalized distance as well.

### Algorithm

In the rest of the section, we give a practical algorithm, i.e., Algorithm 1, to minimize the NN loss function, which is defined as the normalized distance interpreted in (12), i.e.,

(19) $$L\left(\phi ,h_{t},h^{p^{\phi }}\right)≜\frac{∥h^{p^{\phi }}-\frac{h^{p^{\phi }}\cdot h_{t}}{∥h_{t}{∥}^{2}}h_{t}∥}{∥\frac{h^{p^{\phi }}\cdot h_{t}}{∥h_{t}{∥}^{2}}h_{t}∥}.$$

**Figure 1:**
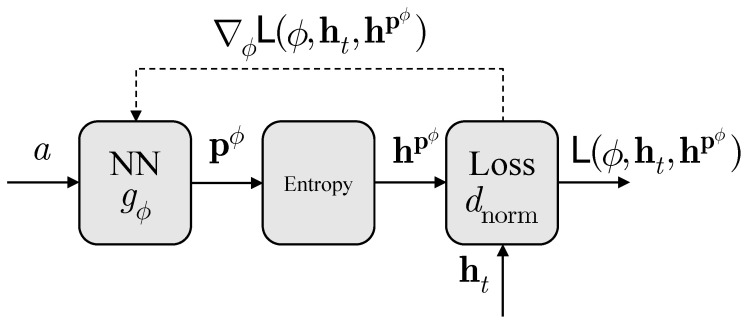
The overall architecture of the proposed method. The dashed arrow indicates the flow of gradients in back-propagation.

Algorithm 1 trains parameters ϕ with a gradient descent method to find an NN that achieves $h^{*}$ in Theorem 1, i.e., identify the entropic vector closest to the target and provide the corresponding underlying PMF. The overall architecture of the proposed method is depicted in Figure 1. More specifically, at each iteration, the NN takes a constant as the input, and outputs a joint PMF $p^{\phi }$. By configuring the last layer of NN as the softmax layer in (17), i.e.,

(20) $$p_{j}^{\phi }=\sigma_{\mathrm{sm}}{\left({\tilde{p}}^{\phi }\right)}_{j}=\frac{\exp({\tilde{p}}_{j}^{\phi })}{\sum_{j=1}^{m}\exp\left({\tilde{p}}_{j}^{\phi }\right)},\;j=1,2,\ldots ,m,$$

where ${\tilde{p}}^{\phi }\in R^{m}$ is the layer input, the resulting PMF $p^{\phi }$ is valid in $\Delta_{X}^{m}$. Subsequently, the associated entropic vector $h^{p^{\phi }}$ is computed from $p^{\phi }$ with (2), (3), and (5). The normalized distance between the associated entropic vector and target vector is then evaluated with (19). To optimize NN parameters ϕ, the gradient descent method is performed in turn. The training procedures are iterated for a sufficiently large number *N*.
**Algorithm 1:** Optimize PMFs for the associated entropic vector closest to the target vector $h_{t}\in \Gamma_{n,X}$ via NN training
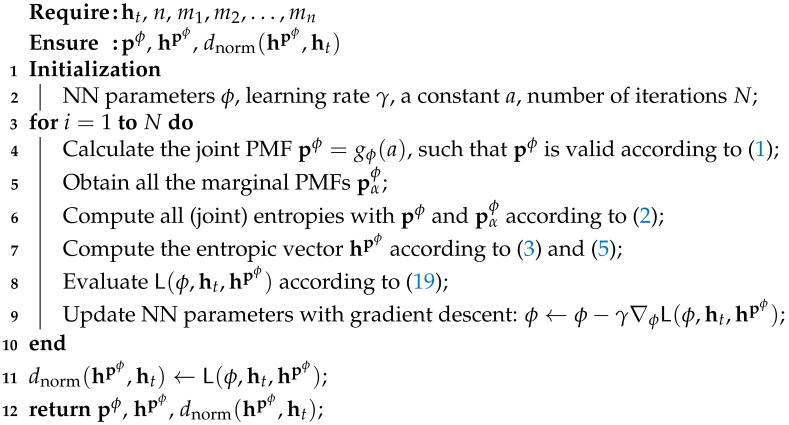


We further implement Algorithm 1 and the NN model in Figure 1 with CNNs, and details are presented in Section 4. With the proposed implementation techniques, the convergence performances of Algorithm 1 for different targets are demonstrated in Section 5.1. Other empirical results on various problems related to the entropic region are demonstrated in Section 5.2 and Section 5.3.

Before ending this section, in the following remark, we discuss the connections between our work with existing literature [44,45,46], which focus on the neural optimization of distributions for problems in information theory as well.

*Remark:* Recently, the optimization of probability distributions utilizing generative NNs in information theory has been studied and applied to plenty of problems in [44,45,46]. In [44,46], NNs are designed to generate the input distributions of given channels, and optimized to produce a distribution that achieves channel capacity, serving as a part of the joint estimation–optimization architecture for channel capacity. More specifically, over continuous spaces [44], a model named neural distribution transformer (NDT) is proposed; the optimized NDT transfers uniformly distributed samples into data that are distributed according to the desired capacity-achieving distribution. Over discrete alphabets [46], the PMF generator is proposed to optimize the PMF numerically, and capacity-achieving channel input data can be sampled from the optimized PMF. We note that [46] focuses on channels with feedback and memory, and the PMF generator is based on a time-series deep reinforcement learning model. For channels without feedback and memory, the PMF generator in [46] degenerates to a similar architecture as the one proposed in this paper. However, different from sampling data from generated PMFs, the method proposed in this paper directly computes entropic vectors from PMFs, and theoretical guarantees are provided in the context of this task. In [45], NNs are also used to optimize conditional distributions that achieve the rate-distortion function for any given source distribution, in both continuous and discrete spaces. The PMF generator in [45] takes source samples as input, and produces corresponding conditional PMFs. If the source is deterministic in [45], the conditional PMF generator degenerates to the proposed model in this paper as well.

## 4. Implementation

In this section, we aim to present implementation details of Algorithm 1 and the NN model in Figure 1 with more advanced deep learning techniques. In addition, we demonstrate that the implementation results in significantly larger feasible alphabet sizes for Algorithm 1 than existing methods, which will contribute to constructing achievable schemes for network coding problems, as discussed in Section 6.

### 4.1. Implementing with Convolutional Neural Networks

We recall that, for a random vector X, if we assume $|X_{i}|=k_{i}=k,i\in N_{n}$ with joint PMFs $p\in \Delta_{X}^{k^{n}}$, then every PMF is a $k^{n}$-dimensional vector. In addition, we notice that Algorithm 1 and the model depicted in Figure 1 require the dimension of the output of the NN to match the dimension of the desired PMF. Hence, the size of the last layer of the NN grows polynomially with the alphabet size *k* of each random variable, and grows exponentially with the number of random variables *n*. Moreover, the indexing problem to calculate all marginal PMFs in Algorithm 1 is computational expensive as well with large dimensional PMF vectors.

Therefore, we are motivated to implement Algorithm 1 and the model in Figure 1 with CNNs, which transfer the vector data in NNs to tensors. Furthermore, by virtue of high-performance GPUs, the training of CNNs can be notably accelerated.

More specifically, if we focus on the case when n=4, a PMF p for X with $|X_{i}|=k,i\in N_{n}$ is a $k^{4}$-dimensional vector such that $p\in \Delta_{X}^{k^{4}}$. To implement with CNNs, we naturally utilize the 3d-CNNs (available in PyTorch [59]), whose data in each layer is a four-dimensional cube. By modifying the NN in Figure 1 with 3d-CNNs, the model takes a constant tensor A as input, where $A\in R^{1\times 1\times 1\times 1}$, and outputs a PMF tensor $P^{\phi }\in {\left[0,1\right]}^{k\times k\times k\times k}$.

We demonstrate an example of a configuration of the modified model in Table 1. Our empirical results in Section 4.2 show that the model implemented with a CNN is feasible even when *k* is large, acquiring additional gain from GPU acceleration.

### 4.2. Feasibility for Large Alphabets

**Figure 2:**
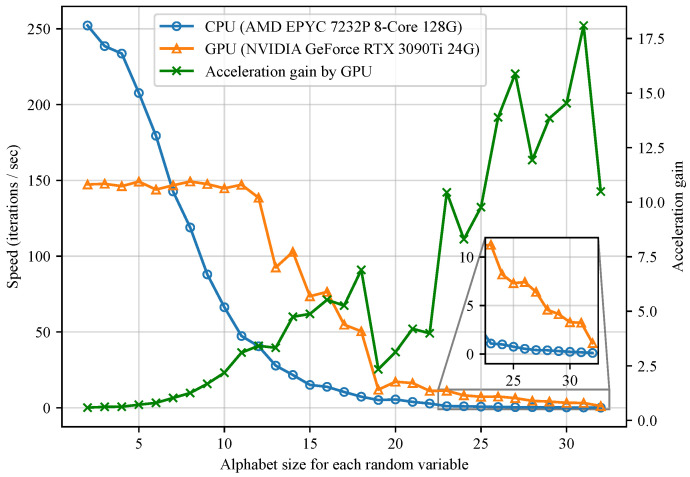
Empirical results on the speed of Algorithm 1 implemented with CNNs. When the alphabet size for every random variable grows from 2 to 32, the iteration speeds and acceleration gains are shown, respectively.

In the proposed method, despite the fact that the complexity of the output layer in the original NN grows as the alphabet sizes of the random variables increase, after the implementation discussed in Section 4.1, the training of CNNs can be accelerated by high-performance GPUs, resulting in significant gains in the training speed for Algorithm 1. In Figure 2, we present empirical results of the training speed of the proposed method as the alphabet sizes of random variables increase.

To the best of the authors’ knowledge, when n=4, the feasible alphabet size of the proposed method (i.e., 32 for every random variable) is larger than prior works. For instance, the largest alphabet size considered in [26] is 5. Alphabet sizes of 10 and 11 are mentioned in [16,21], respectively.

## 5. Empirical Results

In this section, empirical results of the method proposed in Section 3 are presented, focusing on various problems related to $\Gamma_{4}^{*}$ and ${\bar{\Gamma }}_{4}^{*}$. Firstly, we compare our method with the existing random search algorithm [26]. Secondly, we tackle the problems of optimizing the Ingleton score and Ingleton violation index. Lastly, we exploit the proposed method to obtain many entropic vectors that approximate the boundary of $\Gamma_{4}^{*}$, and use the convex hull of these entropic vectors to form an inner bound of ${\bar{\Gamma }}_{4}^{*}$.

Before presenting these results, we first provide a brief introduction of regions within $H_{4}$. When n=4, the Ingleton inequality [41] plays an essential role in the characterization of ${\bar{\Gamma }}_{4}^{*}$, and one permutation instance of the Ingleton inequality is defined as follows.

**Definition** **5** (Ingleton inequality)**.** *For a vector $h\in H_{4}$, one specific permutation instance of the Ingleton inequality [41] is denoted as the inner product between the coefficients $I_{34}$ and h*

(21) $$\begin{array}{cc} I_{34}h≜ & h_{12}+h_{13}+h_{14}+h_{23}+h_{24}\\ & -h_{1}-h_{2}-h_{34}-h_{123}-h_{124}\geq 0. \end{array}$$

*Permuting $N_{4}$, there exist six instances of the Ingleton inequality in total, which are denoted as $Ih\geq 0,I\in R^{6\times 15}$.*

When n=4, the *linear rank region* is the intersection of $H_{4}$ with Ih≥0 (all six instances of Ingleton inequality), i.e.,

(22) $$\Gamma_{4}^{\mathrm{linear}}≜\left\{h\in H_{4}|\;Ih\geq 0\right\}.$$

We consider vectors that represent rank functions of linear subspaces (i.e., linearly representable entropic vectors, or entropic vectors that yield rate regions achieved by linear network codes); then, the linear rank region is the closure of the linear hull of such vectors. The region $\Gamma_{4}^{\mathrm{linear}}$ is a polyhedral convex cone, and is a tight linear inner bound of ${\bar{\Gamma }}_{4}^{*}$ [8,13,60]. However, $\Gamma_{n}^{\mathrm{linear}}$ is only completely characterized for n≤5 [15,17]. Nevertheless, when n=4, the relation $\Gamma_{4}^{\mathrm{linear}}\subset {\bar{\Gamma }}_{4}^{*}\subset \Gamma_{4}$ holds.

Hence, one is interested in characterizing the part of ${\bar{\Gamma }}_{4}^{*}$ excluding $\Gamma_{4}^{\mathrm{linear}}$ (i.e., the almost-entropic vectors that cannot be linearly representable or yield rate regions, which cannot be achieved by linear network codes). We recall that Ih≥0 represents all six instances of the Ingleton inequality. We define

(23) $$\Lambda_{4}≜\left\{h\in \Gamma_{4}|\;Ih\geq 0\;\mathrm{does}\;\mathrm{not}\;\mathrm{hold}\right\},$$

and the entropic part of $\Lambda_{4}$, i.e.,

(24) $$\Lambda_{4}^{*}≜\left\{h\in \Gamma_{4}^{*}|Ih\geq 0\;\mathrm{does}\;\mathrm{not}\;\mathrm{hold}\right\}.$$

We denote the closure of $\Lambda_{4}^{*}$ as ${\bar{\Lambda }}_{4}^{*}$; then, it is clear that $\Lambda_{4}^{*}\subset {\bar{\Lambda }}_{4}^{*}\subset \Lambda_{4}$. By [60] (Lemma 4), any ray in $\Lambda_{4}$ exactly violates one instance of the Ingleton inequality, i.e., $\Lambda_{4}$ is the union of six symmetric regions, each region corresponds to and violates one instance of the Ingleton inequality, and the six regions are disjointed and share boundary points only. Thus, we may consider $\Lambda_{4}$ and $\Lambda_{4}^{*}$ when only one instance of the Ingleton inequality does not hold. More specifically, when $I_{34}h\leq 0$, i.e., the reversed version of (21) holds, we denote $\Lambda_{4}$ and $\Lambda_{4}^{*}$ as $\Lambda_{4}^{34}$ and $\Lambda_{4}^{*,34}$, respectively. Analogously, we can consider different instances of the Ingleton inequality to obtain other five outer regions symmetric to $\Lambda_{4}^{34}$ and five regions symmetric to $\Lambda_{4}^{*}$.

We denote the closure of $\Lambda_{4}^{*,34}$ as ${\bar{\Lambda }}_{4}^{*,34}$; then, in a word, the characterization of ${\bar{\Gamma }}_{4}^{*}$ is reduced to ${\bar{\Lambda }}_{4}^{*,34}$.

The only extreme ray of $\Lambda_{4}^{34}$ violating $I_{34}h\geq 0$ (as well as the Zhang–Yeung inequality [8]) is denoted as $R_{v}$, where

(25) $$v={\left(1,1,1,1,1.5,1.5,1.5,1.5,1.5,2,2,2,2,2,2\right)}^{T}.$$

The region $\Lambda_{4}^{34}$ is often called the *pyramid* and is represented by 15 extreme rays, with $R_{v}$ as the top and the other 14 extreme rays as bases (a complete list of these extreme rays is available in [16] (Section XI)).

In the rest of this section, we focus on the bounded regions as Definition 2 illustrates with $m=k^{4}$, i.e., each random variable has an equal alphabet size *k*. The above discussions still hold, as we denote the bounded versions as $\Lambda_{4,X}^{34}$, $\Lambda_{4,X}^{*,34}$ and ${\bar{\Lambda }}_{4,X}^{*,34}$.

In the following remark, we present the implementation details of Algorithm 1 for experiments in this section.

**Remark** **1.** *In this section, we utilized Algorithm 1 with hyperparameters $n=4,m_{1}=m_{2}=m_{3}=m_{4}=4$. Overall, the NN model followed from the configuration presented in Table 1 with k=4 and either one or three hidden layers. The NN parameters set ϕ was randomly initialized with the default method of PyTorch [59] (the initial parameters can be fixed by fixing the random seed), the optimizer was selected with Adam optimizer [61] with a learning rate γ tuned in the range of $10^{-2}$ to $10^{-5}$ (based on the number of hidden layers), and we selected the constant a=1. The number of iterations N were tuned to be $5\times 10^{4}$ (however, the algorithm can converge with significantly smaller iterations in most tasks of this section).*

### 5.1. Comparison with the Random Search Algorithm in [26]

When evaluating the performance of our method, the target was set as v in (25), which is trivially *not entropic* due to the Zhang–Yeung inequality [8], i.e., $D^{*}>0$ in Theorem 1. We aimed to compare the returned normalized distance by Algorithm 1 with that obtained by the random search algorithm in [26], which is the only existing method for verifying entropic vectors with general form of PMFs. Intuitively, if Algorithm 1 is capable of obtaining entropic vectors closer to v, we are able to approximate finer boundaries of $\Gamma_{4,X}^{*}$.

**Figure 3:**
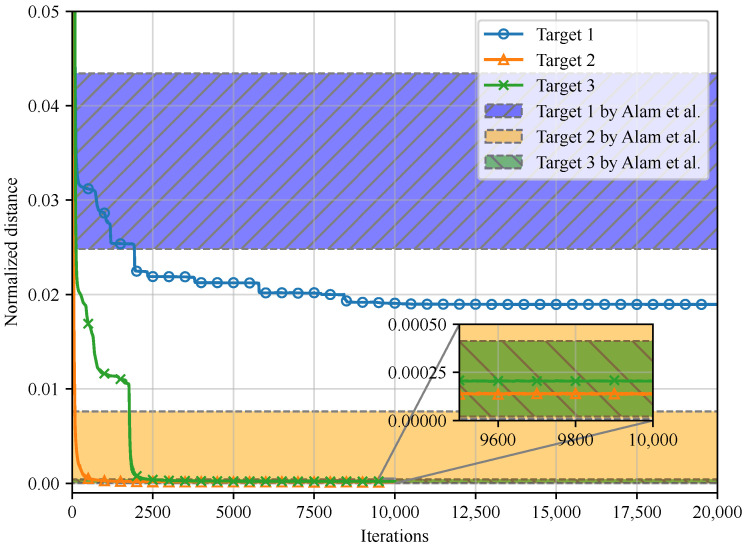
Minimization of normalized distances for different target vectors, compared with the random search algorithm by Alam et al. in [26].

Figure 3 depicts the empirical results of our method. In addition to v (Target 1), two *entropic* vectors in [13] (Theorem 4, Target 2) and [16] (Conjecture 1, Target 3), which belong to the region $\Lambda_{4,X}^{*,34}$, are set as targets, and the results are compared to [26] (the numerical data of the results in [26] are available in [62]). The alphabet size of every random variable, i.e., *k*, is set to 4. In Figure 3, the curves illustrate the convergence performances as the iterations of Algorithm 1 grow, while the shaded regions represent the range of final results obtained by several experiments in [26].

From Figure 3, it is evident that our method outperforms the random search algorithm proposed by [26]. More specifically, for the non-entropic target (Target 1 in Figure 3), the returned normalized distance using our method (0.01895) is smaller than the smallest value obtained by [26] (0.02482). For entropic targets (Targets 2 and 3 in Figure 3), our method returns negligible normalized distance as expected, thus successfully verifying these entropic targets. In addition, the normalized distances obtained by [26] vary across different experiments, and even increase with larger iterations. For example, with Target 1 in Figure 3, the method in [26] returns a normalized distance of 0.02482 with 1226 iterations; however, with 10,091 iterations, a larger normalized distance 0.04283 is obtained [26] (Figure 1).

In contrast, the results obtained by our method are consistent within multiple experiments, empirically presenting better convergence performances than [26].

### 5.2. Optimizing Ingleton Score and Ingleton Violation Index

When optimizing entropic vectors for n=4, it is of great interest to optimize the *Ingleton score* and *Ingleton violation index*. The Ingleton score was first proposed in [42] (Conjecture 4.1) and later rigorously defined in [16] (Definition 3).

**Definition** **6** (Ingleton score [16] (Definition 3))**.** *Given a random vector $X=(X_{1},X_{2},X_{3},X_{4})$, and an entropic vector $h\in \Gamma_{4}^{*}$, the Ingleton score induced by (21) is defined as*

(26) $$I_{34}\left(h\right)≜\frac{I_{34}h}{h_{1234}}.$$

*The Ingleton score is defined as $I≜I_{34}$ due to permutation symmetry, and the optimization problem of I is*

(27) $$\begin{array}{cc} \underset{h}{\inf}\; & I(h),\\ s.t.\; & h\in \Gamma_{4}^{*}. \end{array}$$

Similarly, the Ingleton violation index [19] is defined as follows.

**Definition** **7** (Ingleton violation index [19])**.** *Given a random vector $X=(X_{1},X_{2},X_{3},X_{4})$, and an entropic vector $h\in \Gamma_{4}^{*}$, the Ingleton violation index induced by (21) is defined as*

(28) $$\iota_{34}\left(h\right)≜-\frac{I_{34}h}{∥h∥}.$$

*Due to permutation symmetry, the Ingleton violation index is defined as $\iota ≜\iota_{34}$, and the optimization problem of ι is*

(29) $$\begin{array}{cc} \underset{h}{\sup}\; & \iota (h),\\ s.t.\; & h\in \Gamma_{4}^{*}. \end{array}$$

One is interested in minimizing I or maximizing ι over $\Gamma_{4}^{*}$, because their optimal values reveal the largest violations from (21) among entropic vectors in $\Lambda_{4}^{*,34}$, and, thus, are crucial characterizations of $\Gamma_{4}^{*}$ and ${\bar{\Gamma }}_{4}^{*}$.

There is a rich history of optimizing I [16,21,26,35,42] and ι [19,26]. More specifically, for $h\in {\bar{\Gamma }}_{4}^{*}$, [21] proposes a decomposition and two linear transformations on h. If we denote the composition of these techniques on h as g≜T(h), then it is shown that $g\in {\bar{\Gamma }}_{4}^{*}$, i.e., g is still almost entropic for any almost-entropic h. In addition, the Ingleton score optimized with g in [21] is smaller than the one optimized with h, and refutes the four-atom conjecture in [16] (please refer to [21] (Section VIII) for details). Since T(·) plays an important role in optimizing the Ingleton score and Ingleton violation index in this paper, for both completeness and clarity, we state the details of the techniques that compose T(·) in Appendix A for interested readers.

Thus, we are motivated to exploit our method to obtain a better Ingleton score and Ingleton violation index, when the objective L in Algorithm 1 is replaced by I(h), ι(h), and I(g), ι(g). Although Theorem 1 does not directly imply that the proposed method can be exploited to optimize the Ingleton score and Ingleton violation index, we claim that there always exists an NN that optimizes the Ingleton score and Ingleton violation index to the optimal values to any desired of accuracy as indicated by Theorems 2 and 3. The proofs of Theorems 2 and 3 are provided in Appendix B.2 and Appendix B.3, respectively.

**Theorem** **2.** *For the random vector $X=(X_{1},X_{2},X_{3},X_{4})$ with a fixed and finite alphabet X, we recall the Ingleton score for entropic vectors defined in Definition 6. We consider the entropic vector $h^{*}\in \Gamma_{4,X}^{*}$, such that $h^{*}$ yields the optimal Ingleton score $I^{*}$. Then, there exists an NN with parameters ϕ∈Φ, such that $h^{p^{\phi }}$ is the associated entropic vector of $p^{\phi }=g_{\phi }\left(a\right)$ valid in $\Delta_{X}^{m}$, and $h^{p^{\phi }}$ yields the corresponding Ingleton score $I^{\phi }$, which is consistent with $I^{*}$ within any desired degree of accuracy κ>0, i.e.,*

(30) $$|I^{\phi }-I^{*}|<\kappa ,$$

*where |·| is the absolute value for scalars.*

**Theorem** **3.** *For the random vector $X=(X_{1},X_{2},X_{3},X_{4})$ with a fixed and finite alphabet X, we recall the Ingleton violation index for entropic vectors defined in Definition 7. We consider the entropic vector $h^{*}\in \Gamma_{4,X}^{*}$, such that $h^{*}$ yields the optimal Ingleton violation index $\iota^{*}$. Then, there exists an NN with parameters ϕ∈Φ, such that $h^{p^{\phi }}$ is the associated entropic vector of $p^{\phi }=g_{\phi }\left(a\right)$ valid in $\Delta_{X}^{m}$, and $h^{p^{\phi }}$ yields the corresponding Ingleton violation index $\iota^{\phi }$, which is consistent with $\iota^{*}$ within any desired degree of accuracy μ>0, i.e.,*

(31) $$|\iota^{\phi }-\iota^{*}|<\mu ,$$

*where |·| is the absolute value for scalars.*

The optimized numerical results are compared with the state-of-the-art values in Table 2, and the alphabet size of every random variable is set to 4. In Table 2, our results are rounded to ten decimal digits, and the last four digits are significant since the perturbations for small probability values may lead to large absolute errors of entropy functions and score results.

As seen in Table 2, firstly, for the Ingleton score I, the upper bound −0.0925001031, which is optimized with g, is returned (with negligible improvement compared to −0.0925000777 due to numerical stability). After the first discovery of this value [34,35], the method in [26] obtained a close value of −0.092499 as well, and, thus, we reconfirm this upper bound for the third time. Secondly, for the Ingleton violation index ι, we particularly optimize it with g as well, and a new lower bound 0.0288304 is obtained, which beats the current best value of 0.0281316. We observe that ι actually measures the sine of the angle between the hyperplane $I_{34}h=0$ and h; thus, the obtained new lower bound finds an entropic vector with a larger violation angle from the Ingleton hyperplane, bringing us closer to the optimal ι and the complete characterization of $\Gamma_{4}^{*}$ and ${\bar{\Gamma }}_{4}^{*}$.

**Figure 4:**
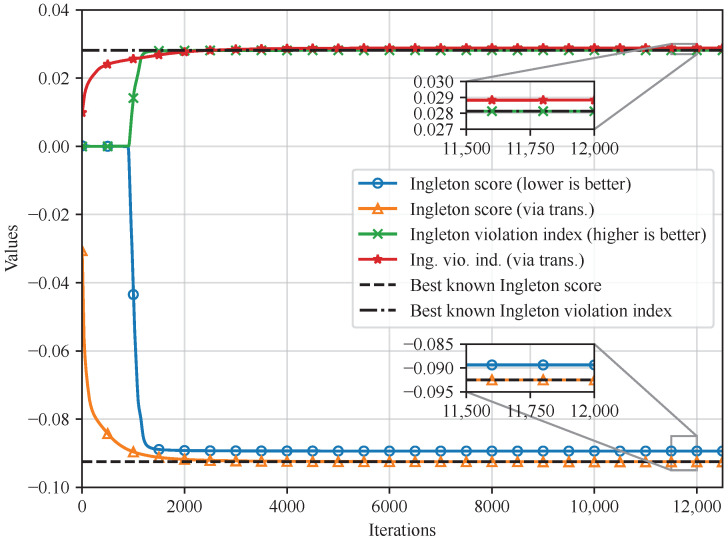
The convergence results on the optimization of the Ingleton score and the Ingleton violation index, where “via trans.” refers to what we optimized with a series of transformations g=T(h) proposed by [21].

The corresponding convergence performances for results in Table 2 are presented in Figure 4. From Figure 4, one can verify the obtained results listed in Table 2, and observe that the proposed method successfully converges to the best known Ingleton score when optimizing I(g), and converges to the Ingleton violation index, which exceeds the best-known results when optimizing ι(g).

**Remark** **2.** *The pursuit of the Ingleton score −0.0925 appears to be difficult with no alternative strategies in the literature. In [26], the search area is constrained using a strategy called hyperplane score reduction with the minimization of normalized distance with relatively large iterations. Additionally, [35] reports that most experiments yield the value −0.09103635, while returning −0.0925000777 very occasionally, even with millions of searches with massive computation resources in parallel. Similarly, with our method, the direct minimization of I(g) yields the value −0.09103635. However, by selecting the entropic point from [13] as the initial point of Algorithm 1 (by setting it as the target and minimizing the normalized distance first), we reproduced the value −0.0925 in a single experiment, with less than 10,000 iterations (which approximately take 6 s).*

### 5.3. Inner Bounding the Almost-Entropic Region

The complete characterization of ${\bar{\Gamma }}_{4}^{*}$ is difficult and open. However, as we find many entropic vectors, a linear inner bound is immediately obtained. For example, for an entropic vector $h\in \Lambda_{4,X}^{*,34}$, if we regard h as the top vertex, then the convex hull of the top h and other 14 base vertices (refer to [16] (Section XI) for a complete list of these extreme rays) yields an inner bound for ${\bar{\Lambda }}_{4,X}^{*,34}$ and ${\bar{\Gamma }}_{4}^{*}$.

In this paper, following [16] (Section XI), the quality of the inner bound is evaluated by the volume ratio. Let ${\tilde{\Lambda }}_{4,X}^{34}$ denote the numerical inner bound obtained, and vol(·) denote the polytope volume computation operation; the volume ratio is defined as

(32) $$R≜\frac{\mathrm{vol}({\tilde{\Lambda }}_{4,X}^{34})}{\mathrm{vol}(\Lambda_{4,X}^{34})}\times 100\%.$$

Intuitively, h needs to be closer to the boundary of $\Lambda_{4,X}^{*,34}$ to obtain a larger volume ratio *R*.

Because Algorithm 1 easily produces entropic vectors with large I and better ι, we are inspired to exploit it to develop a new algorithm, i.e., Algorithm 2, to construct an inner bound with a larger volume ratio, by optimizing entropic vectors that violate other specific hyperplanes.
**Algorithm 2:** Construct inner bounds for the almost-entropic region with Algorithm 1
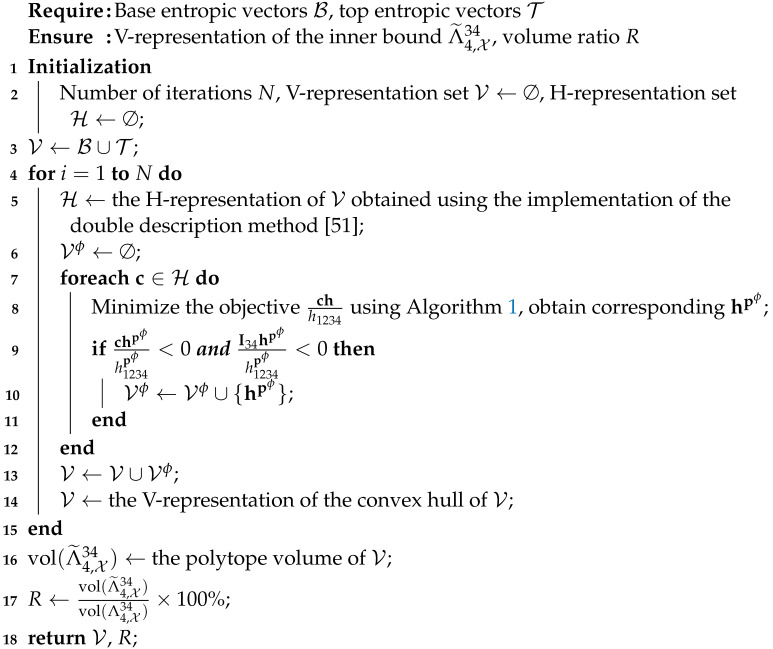


Algorithm 2 can be interpreted as follows. First, starting from the 14 base entropic vectors of $\Lambda_{4,X}^{34}$, i.e., B, we construct an initial inner bound with (an) optimized entropic vector(s), i.e., T, the initial inner bound can be represented by the set of vertices V=B∪T. By transferring the V-representation of the initial inner bound with H-representation, we obtain many hyperplanes with the set of coefficients H. Then, for each of these hyperplanes, we minimize their violation score similar to the Ingleton score using Algorithm 2, i.e., we find entropic vectors $V^{\phi }$ with large violations from the initial inner bound (violating $I_{34}h\geq 0$ as well). Now, we have extended the vertices of the initial inner bound from V to $V\cup V^{\phi }$, and expanded the initial inner bound to an inner bound with a larger volume ratio. This procedure can be repeated for inner bounds with larger volume ratios.

**Remark** **3.** *Since ${∥c∥}_{2}<\infty$, by Theorem 2 and its proof in Appendix B.2, the theoretical guarantee for Algorithm 2, i.e., minimizing the hyperplane score $\frac{ch}{h_{1234}}$ exploiting Algorithm 1, immediately follows.*

We present the volume ratio of the inner bound obtained with Algorithm 2 in Table 3, comparing it with all existing results. For reference, the volume ratio of the outer bound obtained with the non-Shannon-type inequalities in [16] (Section VII) (not tight) was 96.4682%, and the volume of the trivial inner bound obtained with $I_{34}h\geq 0$ was 0. From Table 3, one can see that the volume ratio of the inner bound obtained by our method is larger than existing results. Comparing with the state-of-the-art result [26], the ratio 62.4774% is improved to 72.0437%, leading to the current best inner bound of ${\bar{\Gamma }}_{4}^{*}$ measured by volume ratio. Thus, our proposed method takes another step forward in the complete characterization of ${\bar{\Gamma }}_{4}^{*}$.

**Remark** **4.** *Here, we give details on how we obtained the two results listed in Table 3. For the 66.1340% result, the set T consisted of three entropic vectors that yielded the Ingleton score −0.0925001031, the Ingleton violation indices 0.028131653 and 0.0288304141 in Table 2, respectively, and the entropic vector that yielded the Ingleton score −0.09103635, as discussed in Remark 2. Furthermore, after N=1 iteration of Algorithm 2, we obtained 152 vertices. After the convex hull operation, there were 121 entropic vectors, which gave us an inner bound with a volume ratio of 66.1340%. For the 72.0437% result, the set T consisted of one entropic vector that yielded the Ingleton score −0.0925001031 in Table 2. Furthermore, after N=2 iterations of Algorithm 2, we obtained 2585 vertices, and this inner bound yielded an estimated (as illustrated in Remark 5) volume ratio of 72.0437%.*

**Remark** **5.** *There are several algorithms available for polytope volume computation, as listed in [63]; we chose “Qhull” [64,65] and “lrs” [66,67], which only require the V-representation of a polytope, while providing the convex hull operation as well. However, all existing numerical methods become computationally intractable with high dimension and a large number of vertices (details of their computation complexities can be found in [65,67]). In our case, “Qhull” was able to compute the result 66.1340% from 152 entropic vectors in $R^{15}$, but became intractable when computing another result where the number of entropic vectors was 2585. Nevertheless, “lrs” provides a function called “Estimate”, which allowed us to estimate the computational time and volume result, without diving into the complete computation process. In this manner, it is estimated that the volume ratio computed from 2585 entropic vectors is 72.0437%. The estimation process took several days, and it is suggested that the complete computation will take more than one year.*

## 6. Conclusions and Discussion

This paper introduced a novel architecture to parameterize and optimize PMFs to verify entropic vectors with NNs. Given a target vector, an algorithm is proposed to identify the entropic vector closest to it via NN training. Empirical results demonstrate smaller normalized distances, and improved convergence performances. Optimized Ingleton scores are presented, and a new lower bound on the Ingleton violation index was obtained. A state-of-the-art inner bound of ${\bar{\Gamma }}_{4}^{*}$ was constructed, which was measured by the volume ratio. However, there exist computation burdens in the proposed algorithms. Although Algorithm 1 achieves a larger feasible alphabet size than previous works, its efficiency is still constrained by the alphabet size, as Figure 2 shows. Algorithm 2, which requires auxiliary algorithms to calculate the polytope volume, is limited by the auxiliary algorithms due to high dimensionality and the large number of entropic vectors, as Remark 5 discusses.

Future work includes developing *a computer-aided approach to construct achievable schemes for network coding problems* using the proposed method. The *Linear Programming bound* [68], which yields Shannon outer bounds for rate regions of network coding problems, is not tight in general due to $\Gamma_{n}\neq {\bar{\Gamma }}_{n}^{*}$. However, with Algorithm 1, the corner points obtained by the Linear Programming bound can be verified to be entropic or not. Furthermore, if a corner point is verified to be entropic, the returned PMF indicates the coding scheme. Similar arguments have been raised in [26] as well, where the network instances are simple, and the largest alphabet size is small. Nevertheless, due to smaller and more consistent normalized distances depicted in Figure 3 and the feasibility for large alphabets discussed in Section 4.2, we believe that our proposed method can be applied to network coding problems with larger alphabet sizes, and/or more random variables.

## Figures and Tables

**Table 1:** An example of a configuration when the NN in Figure 1 is implemented with a CNN.

Layer	Output Dimension	Kernel Size	Padding	Activation Function
Input	1×1×1×1	-	-	-
Conv. 3d	k×k×k×k	k×k×k	k−1	ELU
Conv. 3d	k×k×k×k	3×3×3	1	ELU
Conv. 3d	k×k×k×k	3×3×3	1	ELU
Flatten	$k^{4}$	-	-	softmax
Output	k×k×k×k	-	-	-

**Table 2:** Optimized Ingleton scores and Ingleton violation indices.

Objective	Best-Known Results	Our Results
$\inf_{h}I\left(h\right)$	−0.089373 [16]	−0.0893733002
$\inf_{h}I\left(g\right)$	−0.0925000777 [35]	−0.0925001031
$\sup_{h}\iota \left(h\right)$	0.028131604 [26]	0.0281316527
$\sup_{h}\iota \left(g\right)$	N/A	0.0288304141

**Table 3:** Volume ratios of inner bounds of ${\bar{\Lambda }}_{4,X}^{*,34}$.

Methods	Volume Ratio *R* (%)
Newton’s method [16]	53.4815
Non-isomorphic supports [22]	57.8
Grid approach [26]	62.4774
Ours ^1^	66.1340
	72.0437

^1^ For details of the results, refer to Remarks 4 and 5.

## Data Availability

The original contributions presented in the study are included in the article, further inquiries can be directed to the corresponding author.

## Appendix A. The Decomposition and Linear Transformations Proposed in [21]

In [21], a decomposition and two linear transformations on a given entropic vector are proposed to obtain another entropic vector that yields a better Ingleton score than the four-atom conjecture in [16]. Here, we give an introduction to these techniques.

More specifically, for any vector $h\in \Gamma_{n}$, it is decomposed into two parts, i.e., the *modular* part $h^{\mathrm{mod}}$ and the *tight* part $h^{\mathrm{ti}}$, where the element of $h^{\mathrm{mod}}$ is

(A1) $$h_{\alpha }^{\mathrm{mod}}≜\sum_{i\in \alpha }\left(h_{N_{n}}-h_{N_{n}\setminus \left\{i\right\}}\right),\;\forall \alpha \subseteq N_{n}\setminus \emptyset ,$$

and the element of $h^{\mathrm{ti}}$ is

(A2) $$h_{\alpha }^{\mathrm{ti}}≜h_{\alpha }-\sum_{i\in \alpha }\left(h_{N_{n}}-h_{N_{n}\setminus \left\{i\right\}}\right),\;\forall \alpha \subseteq N_{n}\setminus \emptyset ,$$

such that $h=h^{\mathrm{mod}}+h^{\mathrm{ti}}$. It is shown that, for any $h\in {\bar{\Gamma }}_{n}^{*}$, there is $h^{\mathrm{ti}}\in {\bar{\Gamma }}_{n}^{*}$ [21] (Section III).

Moreover, two linear transformations on vectors in $H_{4}$ are proposed. We first introduce notations to define these mappings. For any $h\in \Gamma_{n}$, it satisfies the submodularity of polymatroidal axioms, i.e., for any $\alpha ,\beta \subseteq N_{n}\setminus \emptyset$, there is $h_{\alpha }+h_{\beta }-h_{\alpha \cup \beta }-h_{\alpha \cap \beta }\geq 0$. We further denote the submodularity as the inner product between the coefficient vector $\Delta_{\alpha ,\beta }$ and the vector h, i.e., $\Delta_{\alpha ,\beta }h\geq 0$. For $\beta \subseteq N_{n}$ and $0\leq s\leq |N_{n}\setminus \beta |,s\in N$, we define the vector $r_{s}^{\beta }$ with the element ${\left(r_{s}^{\beta }\right)}_{\alpha }=\min\{s,|\alpha \setminus \beta |\},\forall \alpha \subseteq N_{n}\setminus \emptyset$. Now, for any $h\in H_{4}$, the two linear transformations proposed in [21] (Section VI) can be defined as

(A3) $$A_{3,4}h≜h+\left(r_{1}^{\left\{3\right\}}-r_{1}^{\emptyset }\right)\left(\Delta_{3,4}h\right),$$

and

(A4) $$B_{34,1}h≜h+\left(r_{2}^{\left\{1\right\}}-r_{3}^{\emptyset }\right)\left(\Delta_{134,234}h\right).$$

Given a vector $h\in {\bar{\Gamma }}_{4}^{*}$, we define $T\left(h\right)≜A_{3,4}B_{34,1}h^{\mathrm{ti}}$, where $h^{ti}$ is the tight part of h as (A2) defines, and $A_{3,4}B_{34,1}$ is the composition of the two linear transformations as (A3) and (A4) define. Then, if we denote g≜T(h), it is shown that $g\in {\bar{\Gamma }}_{4}^{*}$, i.e., g is still almost entropic for any almost-entropic h [21] (Section VI).

Please refer to [21] for further details of these techniques.

## Appendix B. Proofs of Theorem 1–3

### Appendix B.1. Proof of Theorem 1

We first define an NN-based mapping which approximates the joint PMF of the random vector X (similar definitions can be found in [45,46]). We define the continuous mapping $g_{\phi }:U\to \Delta_{X}^{m}$. The set U⊂R is defined with |U|≜1 such that a∈U,a<∞, and $\Delta_{X}^{m}$ is the probability simplex of X. The output of the mapping is denoted as $p^{\phi }≜g_{\phi }\left(a\right)$, where $p^{\phi }\in \Delta_{X}^{m}$.

In the defined mapping $g_{\phi }$, the set U is compact and $g_{\phi }$ is continuous. Thus, we can utilize the universal approximation theorem [56] to guarantee that there exists a parameter set ϕ such that the output of NN $p^{\phi }=g_{\phi }\left(a\right)$ is closed to any desired PMF $p^{*}=g^{*}\left(a\right)$. We note that the universal approximation theorem holds regardless of whether $l_{1}$ or $l_{2}$ norm is used. Thus, we have the following statement. For the random vector X with finite alphabet X, given a desired joint PMF $p^{*}\in \Delta_{X}^{m}$ and ϵ>0, there exists an NN with parameters ϕ∈Φ, where $\Phi \subset R^{d}$ is the parameter space, and the parameterized mapping $g_{\phi }\in G^{\left(1,m\right)}$ generates the PMF $p^{\phi }=g_{\phi }\left(a\right)$, such that

(A5) $$∥p^{\phi }-p^{*}{∥}_{1}<\epsilon ,$$

where ${∥\cdot ∥}_{1}$ is the $l_{1}$ norm.

Then, we assume that $h^{*}$ is associated with PMF $p^{*}$; we need to prove that, when $p^{\phi }\to p^{*}$ in $l_{1}$ norm, we have $h^{p^{\phi }}\to h^{*}$ in $l_{2}$ norm.

We consider X with an index set $N_{n}$ and a fixed finite alphabet X; from [54] (Section 2.3), we have $h_{N_{n}}^{p^{\phi }}\to h_{N_{n}}^{*}$ in absolute value, as $p^{\phi }\to p^{*}$ in variational distance [54] (Definition 2.23) (i.e., $∥p^{\phi }-p^{*}{∥}_{1}\to 0$). More specifically, for any $\eta^{\prime }>0$, there exists $\epsilon^{\prime }>0$, such that, for all

(A6) $$∥p^{\phi }-p^{*}{∥}_{1}<\epsilon^{\prime },$$

where ${∥\cdot ∥}_{1}$ is the $l_{1}$ norm, there is

(A7) $$|h_{N_{n}}^{p^{\phi }}-h_{N_{n}}^{*}|<\eta^{\prime },$$

where |·| is the absolute value for scalars.

We note that, for any $\alpha \subseteq N_{n}\setminus \emptyset$, there is $∥p_{\alpha }^{\phi }-p_{\alpha }^{*}{∥}_{1}\leq ∥p^{\phi }-p^{*}{∥}_{1}<\epsilon^{\prime }$ by [26] (Corollary 1), and, thus, we have $|h_{\alpha }^{p^{\phi }}-h_{\alpha }^{*}|\leq |h_{N_{n}}^{p^{\phi }}-h_{N_{n}}^{*}|<\eta^{\prime }$. Consequently, we have

(A8) $$\sum_{\alpha \subseteq N_{n}\setminus \emptyset }|h_{\alpha }^{p^{\phi }}-h_{\alpha }^{*}|=∥h^{p^{\phi }}-h^{*}{∥}_{1}<\left(2^{n}-1\right)\cdot \eta^{\prime },$$

and, by the relationship between $l_{1}$ and $l_{2}$ norm, we have

(A9) $$∥h^{p^{\phi }}-h^{*}∥\leq ∥h^{p^{\phi }}-h^{*}{∥}_{1}<\left(2^{n}-1\right)\cdot \eta^{\prime }.$$

We note that $h^{*}$ yields the minimal normalized distance to $h_{t}$, i.e., there exist $h_{t}^{\prime }\in R_{h_{t}}$ such that $∥h^{*}-h_{t}^{\prime }∥=∥h_{t}^{\prime }∥\cdot D^{*}$ by Definition 3. Then, by the triangular inequality, we have

(A10) $$\begin{array}{cc} ∥h^{p^{\phi }}-h_{t}^{\prime }∥ & \leq ∥h^{p^{\phi }}-h^{*}∥+∥h^{*}-h_{t}^{\prime }∥\\ & <\left(2^{n}-1\right)\cdot \eta^{\prime }+∥h_{t}^{\prime }∥\cdot D^{*}. \end{array}$$

Thus, for any desired degree of accuracy η>0, if we choose $\eta^{\prime }=\frac{∥h_{t}^{\prime }∥}{2^{n}-1}\eta$ and ϵ to be equal to the corresponding $\epsilon^{\prime }$, then, there exists an NN, such that

(A11) $$∥p^{\phi }-p^{*}{∥}_{1}<\epsilon ,$$

and

(A12) $$\frac{∥h^{p^{\phi }}-h_{t}^{\prime }∥}{∥h_{t}^{\prime }∥}=d_{\mathrm{norm}}\left(h^{p^{\phi }},h_{t}\right)<D^{*}+\eta .$$

Hence, the proof of Theorem 1 is complete. □

### Appendix B.2. Proof of Theorem 2

We recall the proof of Theorem 1; we assume that $h^{*}$ is associated with PMF $p^{*}$; the main idea is to bound (30) as $∥p^{\phi }-p^{*}{∥}_{1}<\epsilon$. The proof can be divided into two steps.

In the first step, we need to bound the term $|I_{34}h^{p^{\phi }}-I_{34}h^{*}|$. More specifically, following (A9) of Theorem 1, we have proved that, for any $\eta^{\prime }>0$, there exists $\epsilon^{\prime }>0$, such that, for all $∥p^{\phi }-p^{*}{∥}_{1}<\epsilon^{\prime }$, there is $∥h^{p^{\phi }}-h^{*}∥<\left(2^{n}-1\right)\cdot \eta^{\prime }$. Similarly, if we choose $\eta^{\prime }=\frac{\eta }{2^{3}-1}$ and ϵ to be equal to the corresponding $\epsilon^{\prime }$, then, there exists an NN such that $∥p^{\phi }-p^{*}{∥}_{1}<\epsilon$ and $∥h^{p^{\phi }}-h^{*}∥<\eta$. Then, by the Cauchy–Schwarz inequality, we have

(A13) $$|I_{34}h^{p^{\phi }}-I_{34}h^{*}\left|=\right|I_{34}\left(h^{p^{\phi }}-h^{*}\right)|\leq ∥I_{34}∥\cdot ∥h^{p^{\phi }}-h^{*}∥<∥I_{34}∥\cdot \eta .$$

In the second step, to complete the rest of proof of (30), we need to bound the term $\left|\frac{I_{34}h^{p^{\phi }}}{h_{1234}^{p^{\phi }}}-\frac{I_{34}h^{*}}{h_{1234}^{*}}\right|$. More specifically, by the triangular inequality, we derive that

(A14)|I34hpϕh1234pϕ−I34h∗h1234∗|=|I34hpϕ·(h1234∗−h1234pϕ)+h1234pϕ·(I34hpϕ−I34h∗)h1234pϕ·h1234∗|(A15)≤|I34hpϕ·(h1234∗−h1234pϕ)h1234pϕ·h1234∗|+|h1234pϕ·(I34hpϕ−I34h∗)h1234pϕ·h1234∗|.

We note that, from (A15), following the proof of Theorem 1, in the first term, we already have $|h_{1234}^{*}-h_{1234}^{p^{\phi }}|<\eta^{\prime }$ as $∥p^{\phi }-p^{*}{∥}_{1}<\epsilon^{\prime }$. In the second term, there is $|I_{34}h^{p^{\phi }}-I_{34}h^{*}|<∥I_{34}∥\cdot \eta$ by (A13). Thus, $\left|\frac{I_{34}h^{p^{\phi }}}{h_{1234}^{p^{\phi }}}-\frac{I_{34}h^{*}}{h_{1234}^{*}}\right|$ can be further bounded as

(A16)|I34hpϕh1234pϕ−I34h∗h1234∗|<|I34hpϕh1234pϕ·h1234∗|·η′+∥I34∥|h1234∗|·η(A17)=η|h1234∗||I34hpϕh1234pϕ|·123−1+∥I34∥.

Because for any entropic $h^{p^{\phi }}$ and $h^{*}$, we have $h_{1234}^{p^{\phi }}>0$ and $h_{1234}^{*}>0$, while $|I_{34}h^{p^{\phi }}|<\infty$, $h_{1234}^{p^{\phi }}<\infty$ and $h_{1234}^{*}<\infty$ hold for a fixed and finite alphabet, and $∥I_{34}∥$ is a bounded constant. Thus, for any desired degree of accuracy κ>0, we choose κ to be equal to (A17). Then, there always exists an NN such that

(A18) $$∥p^{\phi }-p^{*}{∥}_{1}<\epsilon ,$$

and

(A19) $$|\frac{I_{34}h^{p^{\phi }}}{h_{1234}^{p^{\phi }}}-\frac{I_{34}h^{*}}{h_{1234}^{*}}|<\kappa .$$

With (A14) and (A19), by the permutation symmetry of the Ingleton inequality and Ingleton score, (30) is immediately proved.

Hence, the proof of Theorem 2 is complete. □

### Appendix B.3. Proof of Theorem 3

Similar to the proof of Theorem 2, we recall the proof of Theorem 1; we assume that $h^{*}$ is associated with PMF $p^{*}$; the main idea is to bound (30) as $∥p^{\phi }-p^{*}{∥}_{1}<\epsilon$. The proof can be divided into two steps.

In the first step, we bound the term $|I_{34}h^{p^{\phi }}-I_{34}h^{*}|$, which is already proved in the first step of the proof of Theorem 3.

In the second step, similar to the proof of Theorem 2, we need to bound $\left|\frac{I_{34}h^{p^{\phi }}}{∥h^{p^{\phi }}∥}-\frac{I_{34}h^{*}}{∥h^{*}∥}\right|$. We first need to bound the term $|∥h^{p^{\phi }}∥-∥h^{*}∥|$. By the triangular inequality and the proof of Theorem 2, if we choose $\eta^{\prime }=\frac{\eta }{2^{3}-1}$ and ϵ to be equal to the corresponding $\epsilon^{\prime }$, we can derive

(A20) $$∥h^{p^{\phi }}∥=∥h^{p^{\phi }}-h^{*}+h^{*}∥\leq ∥h^{p^{\phi }}-h^{*}∥+∥h^{*}∥<\eta +∥h^{*}∥,$$

i.e., $∥h^{p^{\phi }}∥-∥h^{*}∥<\eta$. We can similarly prove that $∥h^{*}∥-∥h^{p^{\phi }}∥<\eta$, thus

(A21) $$-\eta <∥h^{p^{\phi }}∥-∥h^{*}∥<\eta ,$$

and, equivalently,

(A22) $$|∥h^{p^{\phi }}∥-∥h^{*}∥|<\eta .$$

As we have bounded $|∥h^{p^{\phi }}∥-∥h^{*}∥|$, in the rest of the proof, following the same proof strategy as Theorem 3 (i.e., (A14) to (A19)), it is straightforward to prove that, for any desired degree of accuracy μ>0, by properly choosing μ, there always exists an NN such that

(A23) $$∥p^{\phi }-p^{*}{∥}_{1}<\epsilon ,$$

and

(A24) $$|\frac{I_{34}h^{p^{\phi }}}{∥h^{p^{\phi }}∥}-\frac{I_{34}h^{*}}{∥h^{*}∥}|<\mu .$$

With (A23) and (A24), by the permutation symmetry of the Ingleton inequality and Ingleton score, (31) is immediately proved.

Thus, the proof of Theorem 3 is complete. □
